# Incidental Carcinoid Tumor Arising in a Meckel’s Diverticulum

**DOI:** 10.7759/cureus.68562

**Published:** 2024-09-03

**Authors:** Jonah R Duran, Levi J White, Peter M Dunn, Yong S Chang, Andrew J Knauer

**Affiliations:** 1 General Surgery, Touro College of Osteopathic Medicine, Middletown, USA; 2 General Surgery, Garnet Health Medical Center, Middletown, USA

**Keywords:** closed-loop small bowel obstruction, neuroendocrine tumor, meckel’s diverticulum, carcinoid tumor, incidental tumor

## Abstract

This case report describes a patient who presented with concern for a closed-loop small bowel obstruction (SBO). During exploratory laparotomy, an area of ischemic bowel due to closed loop obstruction was resected, along with an incidentally discovered inflamed-appearing Meckel’s diverticulum (MD). The resected specimen contained a well-differentiated carcinoid tumor of benign behavior with a maximum diameter of 0.6 cm, which invaded the submucosal layer (pT1b and pN0). Over the last several years, there has been a debate with little consensus regarding the proper surgical management in the case of an asymptomatic MD that is discovered incidentally during abdominal exploration. The intention of sharing this case is to underline the importance of the decision-making process in treating patients with this intraabdominal pathologic condition found incidentally at the time of surgery.

## Introduction

Embryology

Meckel's diverticulum (MD) is considered the most common congenital anatomic variant of the gastrointestinal (GI) tract, found in 2% of the population [1]. The anatomic variant was initially identified by Fabricus Hildanus in 1598; however, Johann Meckel was the first to publish a detailed description of this finding. During the embryonic stage at three weeks of development, a connection exists between the yolk sac and the primitive gut via a broad vitelline duct. During the eighth week, as the placenta replaces the yolk sac as the source of fetal nutrition, the duct is normally obliterated. Failed or incomplete obliteration of the vitelline (or omphalomesenteric) duct may lead to several possible anomalies, including omphalomesenteric fistula, enterocyst, fibrous band connecting the intestine to the umbilicus, or MD [2]. Around 90% of all vitelline duct anomalies are attributed to MD, whether it is linked to the umbilicus or the mesentery or not [1].

Anatomy

Anatomically, MD is a true diverticulum that consists of all the layers found in the normal intestinal wall. The "rule of twos" is often used to describe the characteristics of MD, which include its frequency of occurrence in 2% of the population, length of approximately two inches, location within two feet of the ileocecal valve, two types of ectopic tissue that are commonly present (mostly gastric and pancreatic), and a higher incidence in males under two years of age [2, 3]. 

While complications associated with MD, such as persistent fistulas or fibrous band formations, are rare, the vast majority of Meckel’s diverticula are asymptomatic, broad-based lesions found incidentally during surgical exploration or radiographic study of the small intestine.

Neuroendocrine tumor (NET) in Meckel’s diverticula

Malignancy is a rare complication associated with MD, estimated to occur in 0.5% to 3.2% of cases. Carcinoid is the most common type of malignancy arising from a MD, accounting for 76.5% of cases [2, 3], but other pathologic types such as adenocarcinoma, pancreatic carcinoma, intraductal papillary mucinous neoplasm, gastrointestinal stromal tumors and leiomyosarcomas, lymphoma, lipoma, adenomyoma, and villous adenoma can also occur [3]. Carcinoid tumors are a distinct type of neuroendocrine neoplasm with characteristic histological, clinical, and biological properties. The clinical presentation and biological features of these tumors are heterogeneous and can range from indolent, unrecognized entities to highly active, metastatic secretory tumors. They can either remain asymptomatic for years, manifest alongside obstructive symptoms, or result in the presence of liver metastases. Jejunoileal NETs originate from the diffuse neuroendocrine system (enterochromaffin cells in the crypts of Lieberkühn), located in the GI tract that may produce serotonin or other neuroamines and peptides that may cause carcinoid syndrome [4]. Carcinoids arising from MD are often found incidentally during an unrelated operation or at autopsy, with over half of patients showing no symptoms. However, in about one-third of cases, the tumor can cause symptoms requiring surgical intervention; the diagnosis is nearly never apparent till the specimen has been removed [5]. Individuals experiencing symptoms commonly describe abdominal discomfort, diarrhea, loss of weight, feelings of nausea, and episodes of vomiting [6]. Obstruction of the small intestine, inflammation, and lower gastrointestinal tract bleeding accounted for 90% of the presenting symptoms [5]. Less than 200 instances of NETs originating from MD have been reported in published literature, with the preponderance of these cases documented within larger series of GI NETs and fewer as standalone case reports [6]. A study by Thirunavukarasu et al. found that a significant proportion of patients with MD malignancies had other primary malignancies at some point during their lifetime [3]. Out of the 158 patients diagnosed with Meckel's diverticulum cancer (MDC), 33% experienced an additional primary malignancy at a location other than the Meckel site at some point in their lives, and in 13% of these patients (n = 7), MDC was the first malignancy to occur.

Due to its rare occurrence, predicting the clinical behavior and outcome of this lesion can be challenging. However, by bringing this to light, we hope to lead to the diminution of the likelihood of inadvertently neglecting these typically benign tumors, which can become metastatic if left untreated. Familiarity with uncommon sites of origin, such as MD, can aid in the accurate identification and characterization of such tumors, leading to prompt and appropriate treatment [6].

## Case presentation

**Figure 1:**
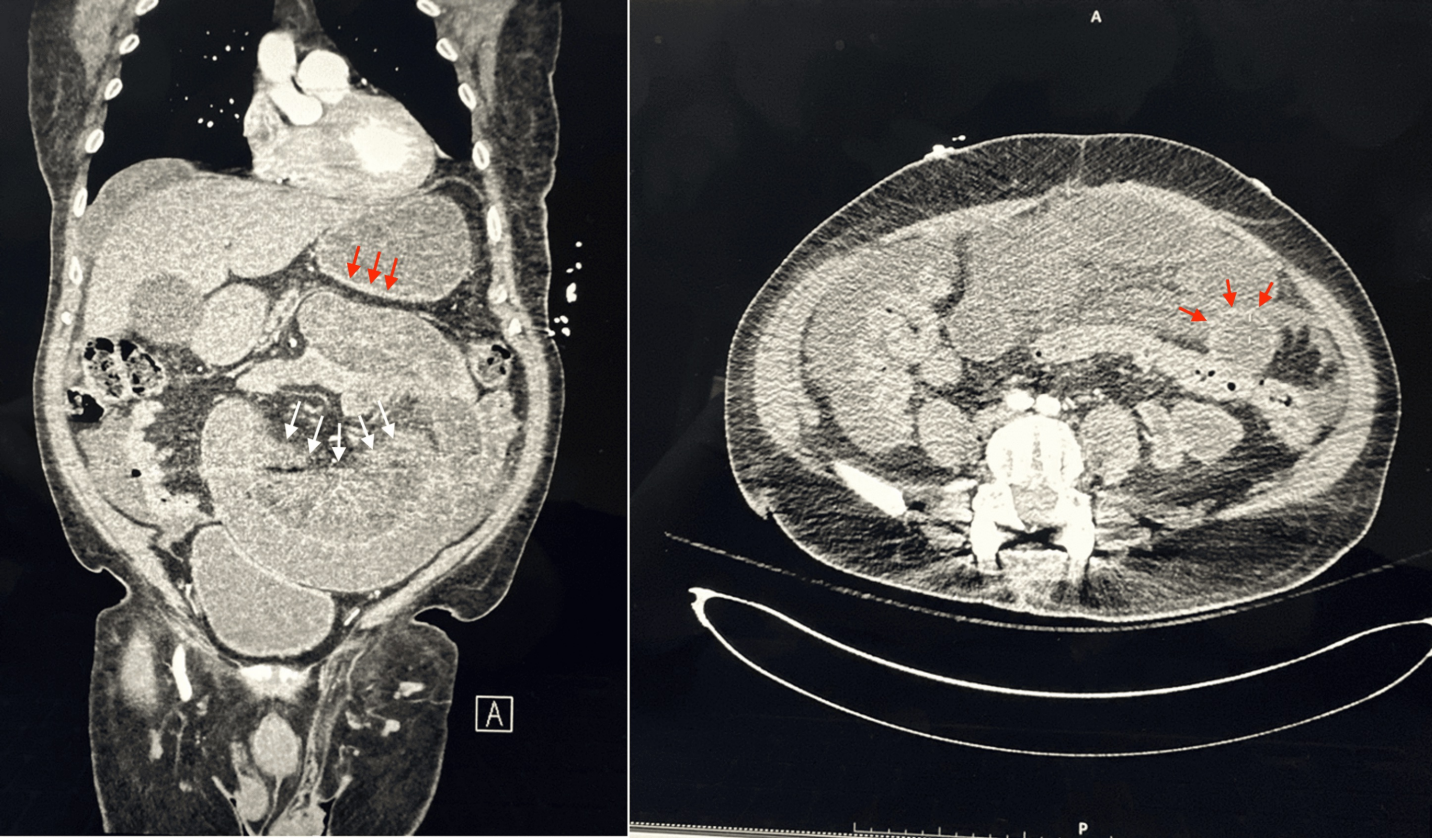
Small bowel obstruction A CT scan of the abdomen and pelvis demonstrates dilated air-filled small bowel loops with distal decompression consistent with typical findings of complete small bowel obstruction, along with thickened and increased attenuation of the bowel wall (red arrows) and mesenteric edema (white arrows) associated with intestinal ischemia.

The patient was a 63-year-old male with a past medical history of end-stage renal disease (ESRD) on hemodialysis (HD), atrial fibrillation, hypertension, hyperlipidemia, and obesity (body mass index 31.1 kg/m^2^). He presented with 10/10 intensity, periumbilical abdominal pain associated with nausea without vomiting, and was admitted for a closed-loop small bowel obstruction (SBO) with suspected bowel or intestinal strangulation. The abdominal pain lasted for half an hour, radiating to the midback, and was described as a constant, sharp pain without modifying factors. The pain started suddenly while lying down watching television, causing him to break into a cold sweat. The emergency medical service personnel noted the patient to be pale, cool, and diaphoretic upon arrival. He otherwise felt well in the preceding days with normal appetite and bowel movements. A 30-pound (lb) unintentional weight loss over the past three months was endorsed but denied rectal bleeding, black stool, constipation, or diarrhea, as well as a history of abdominal hernias or prior abdominal surgeries. Physical examination revealed a large, soft abdomen with marked tenderness in the periumbilical region with guarding. The rest of the physical exam was unremarkable. In the emergency department, a blood pressure of 225/97 mmHg was noted to be present, and a nicardipine drip at 5 mg/hr was subsequently started with a marked reduction in blood pressure. True ischemia was still suspected despite a normal lactic acid level. Computed tomography (CT) of the abdomen and pelvis demonstrated a SBO and mesenteric ischemia (Figure 1), prompting general surgery consultation and a decision that would benefit from urgent intervention. The patient was taken urgently to the operating room for exploratory laparotomy and bowel resection.

Surgical intervention

Our management was similar to that of an appendiceal tumor with negative margins and three negative lymph nodes, requiring no further resection. From the patient presentation, lab work, and imaging outlined prior, the patient was diagnosed with a closed-loop bowel obstruction with ischemia, and surgical intervention was indicated. The anesthesiologist assigned the patient an American Society of Anesthesiology score of three, and the procedure was considered emergent. An exploratory laparotomy and a small bowel resection, including the MD with a primary anastomosis under general endotracheal anesthesia, were performed. During the procedure, significant findings of note were an ischemic and hemorrhagic portion of the jejunum and a small bowel volvulus, and a large amount of murky free fluid was encountered, suctioned, and taken for culture once in the peritoneal cavity. While examining the small bowel, an inflamed-appearing MD was found distal to the obstruction, which was deemed the cause of the small bowel volvulus. The colon, stomach, liver, and gallbladder were examined, and all were without acute pathology. However, some gelatinous material in the pelvis was discovered and sent as a separate specimen. The procedure was performed without complications, the patient tolerated the procedure well, was extubated, and transferred to the post-anesthesia care unit (PACU) in stable condition. 

Pathological features

**Figure 2:**
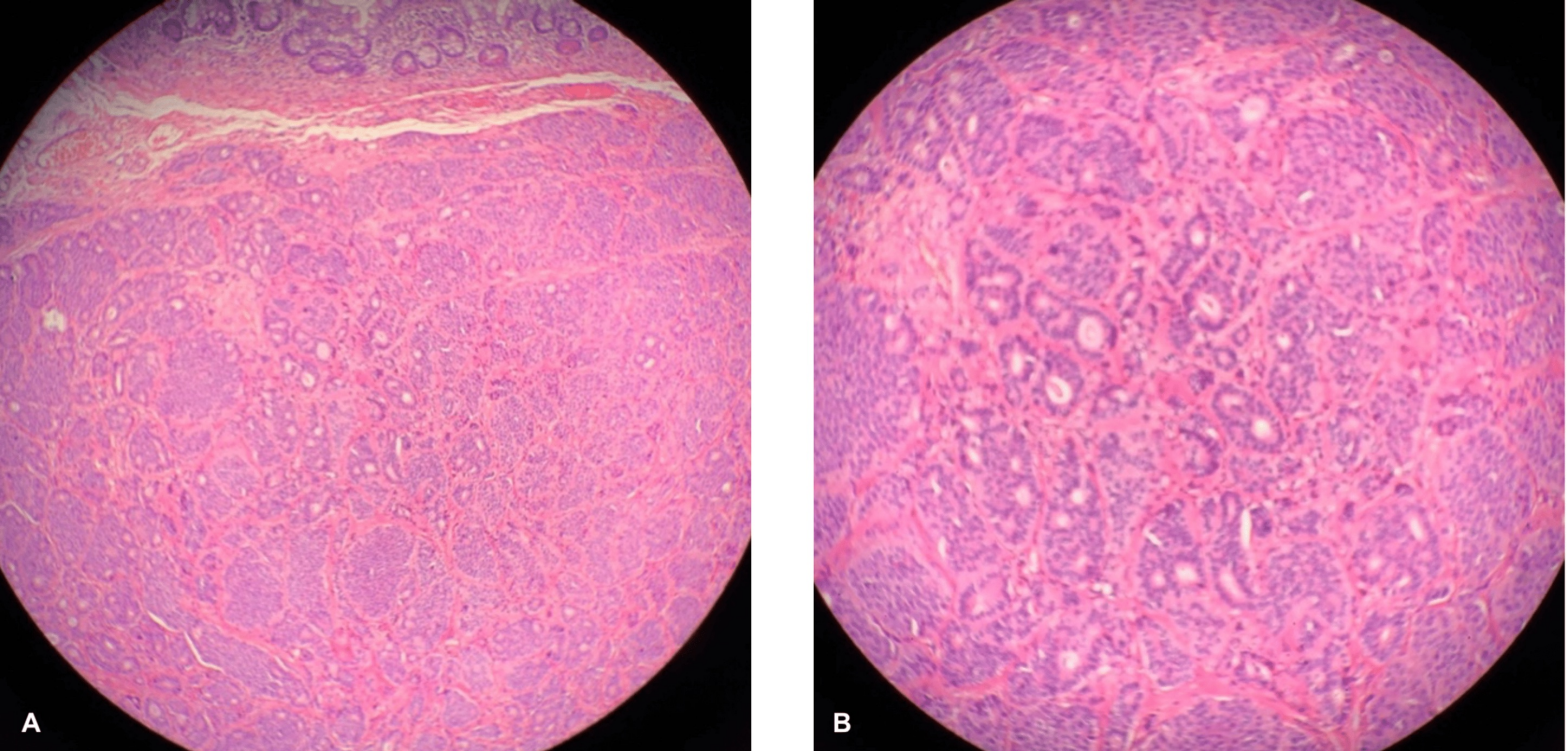
H&E stain (A) Left, 10X magnification of the camera lens: A well-differentiated neuroendocrine tumor demonstrating a nested pattern with relatively uniformly appearing tumor cells under the normal mucosal layer. (B) Right, 40X magnification of the camera lens: Close-up of tumor cells possessing round nuclei with finely stippled "salt and pepper" chromatin. The cytoplasm is moderate and eosinophilic. Mitotic figures are infrequent, with less than two mitoses per 10 high-power fields, and no significant areas of necrosis are observed.

**Figure 3:**
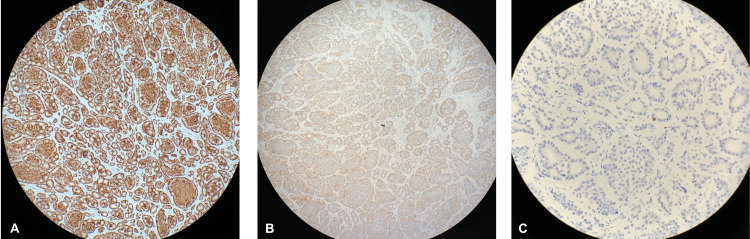
Immunohistochemistry (A) Left, 40X magnification of the camera lens: Chromogranin A immunostaining in neuroendocrine cells. (B) Middle, 20X magnification of the camera lens: Synaptophysin immunostaining in neuroendocrine cells. (C) Right, 40X magnification of the camera lens: Ki-67 positive with an index of less than 2%.

A well-differentiated neuroendocrine carcinoma nodule was found incidentally within the MD at the small intestine. The tumor had a maximum diameter of 0.6 cm arising from the submucosal layer (Table 1). Two of the lymph nodes were examined for potential metastases, but none were involved. All the margins around the tumor were unconcerned by invasive carcinoma, carcinoma in situ, and adenoma. A pathologic primary tumor and regional lymph node were pT1b and pN0, respectively. Hematoxylin and eosin (H&E) staining showed tumor cells forming small glandular features with no indications of necrosis, severe dysplastic changes, or mitotic figures (Figures 2A-2B). Immunohistochemical staining showed the tumor cells that were positive for chromogranin A (Figure 3A), synaptophysin (Figures 3A-3B), and Ki-67 (Figure 3C). MIB-1, an antibody for detecting the Ki-67 antigen with a high sensitivity index, was less than 1%. Histology presented well-differentiated small-cell neuroendocrine carcinoma, NET grade G1.

**Table 1:** Specimen pathology report pTNM: pathological tumor-node-metastasis; AJCC: American Joint Committee on Cancer

Specimen characteristics	
Procedure	Segmental resection
Tumor	
Tumor site	Small intestine, not otherwise specified: tumor noted in Merkel diverticulum
Histologic type	Small cell neuroendocrine carcinoma
Histologic grade	G1: well-differentiated
Tumor size	Greatest dimension (cm) 0.6 cm
Tumor extension	Tumor invades the submucosa
Macroscopic tumor perforation	Not identified
Lymphovascular invasion	Not identified
Margins	
Margins	All margins are uninvolved by invasive carcinoma, carcinoma in situ (high-grade dysplasia), and adenoma
Margins examined	Surgical margin is free of tumor (diverticulum resection)
Closest margin	The tumor is 0.6 cm from the surgical margin of the resection
Lymph nodes	
Number of lymph nodes involved	0
Number of lymph nodes examined	2
Pathologic stage classification (pTNM, AJCC 8th Edition)	
Primary tumor (pT)	pT1b
Regional lymph nodes (pN)	pN0
Additional findings	
Additional findings	Merkel's diverticulum and small bowel focal ischemic change
Comments	
Comments	An incident finding of a well-differentiated neuroendocrine carcinoma nodule arising from the submucosa

## Discussion

Well-differentiated NETs of the small bowel are fairly rare neoplasms that pose several clinical complexities. Numerous cases remain asymptomatic until a late stage, leading to significant impacts. Meckel’s diverticulum, the most common GI tract anomaly, has received a lot of attention recently.

The case presented above outlines an important incidental finding that in turn led to a likely improved outcome for the patient. While presenting symptomatic for an SBO and bowel ischemia, which required surgical intervention, a previously undiagnosed MD was found to be inflamed distal to the site of obstruction, which was deemed the cause of the small bowel volvulus and was included in the small bowel resection with one anastomosis. The MD was postoperatively found to contain a carcinoid tumor that contained free margins on the pathology evaluation of the sample. No further surgery was required at this time, as the patient was expected to have a favorable outcome. To be prudent, a follow-up visit with oncology was recommended to discuss any required further treatment; however, the patient was lost to follow-up.

In recent years, there has been ongoing discussion regarding the appropriate surgical management of asymptomatic MD incidentally identified during abdominal exploration via laparotomy or laparoscopy. A variety of accounts document tumors that have been found incidentally in MD after laparoscopic removal, supporting potential surgical resection of asymptomatic MD, but the lack of clear evidence-based criteria makes it difficult to come to a consensus for proper surgical management. Generally, surgical treatment of carcinoid tumors depends on the size and extent of the primary lesion. In the case of incidentally found non-metastatic, small carcinoids arising in a MD, diverticulectomy is typically performed to obtain a healthy microscopic margin (R0) without requiring small bowel resection [7]. For larger (> 2 cm) or higher-stage lesions, resection of the intestinal segment containing the diverticulum and its mesentery, accompanied by a wide lymphadenectomy up to the level of the superior mesenteric vessel origin, can usually result in a cure [5, 7]. During laparotomy, it is essential to thoroughly examine the entire abdominal cavity, including the entirety of the small intestine, to avoid missing a second localization, which occurs in approximately 30% of cases [7]. Based on recommendations that are outlined by Caracappa et al. [8], the size and clear margins of our tumor warranted no further exploration or surgical intervention to look for metastasis. If this particular patient’s MD did not appear inflamed, it would have been likely left in the abdomen containing a carcinoid tumor that could continue to grow, leading to symptoms as well as potential metastasis to other organs. As a result, the removal of this MD likely led to a better clinical outcome for this patient and should prompt further discussion as to the removal of asymptomatic MDs found incidentally in surgery. The case reported here demonstrates anecdotal support for surgical resection of asymptomatic MD found incidentally at laparotomy, adding to the global conversation regarding what proper surgical management in similar circumstances should be.

In an effort to delineate anatomic criteria for symptomatic MD, a comparison study was conducted by Bani-Hani et al. between incidental and symptomatic cases of MD [9]. The study revealed that the diverticula in the symptomatic group tended to be longer (p = 0.001) with a narrower base (p = 0.001) than the diverticula in the incidental group. A diameter of ≤ 2 cm was significantly associated with more complications (p = 0.01). The symptomatic group had a higher presence of heterotopic tissue than the incidental group (p = 0.01). There was no significant difference in the morbidity rate between the two groups (p = 0.71), and there was no mortality in either group. Meckel’s diverticulum is difficult to diagnose preoperatively and should be considered in cases of acute abdomen. They concluded that resection of incidentally found diverticula does not result in increased operative morbidity or mortality.

Elucidating the metastatic capability of NETs originating from MD is crucial, due to its prognostic implications and its role in guiding treatment approaches. A retrospective study between 1977 and 2009 by Poncet et al. included eight cases of endocrine tumors of MD. Five cases revealed a mesenteric mass or liver metastases; three cases were diagnosed incidentally at laparotomy or laparoscopy. However, due to their rarity and limited data, it is difficult to accurately define their natural history, and the long-term prognosis for patients with Meckel's NETs remains unclear [10]. The metastatic potential of these tumors is debated, with some studies suggesting that they behave aggressively and have a high propensity to metastasize, similar to small bowel NETs. Moyana found that carcinoids of the MD more closely resembled the jejunoileal than appendiceal carcinoids in the immunohistochemical profile and thus may be predicted to behave more aggressively [11]. Although the mean dimensions of NETs within MD resemble appendiceal carcinoids, their inherent progression and biological characteristics are believed to share greater similarities with those emerging from the ileum [4, 7]. Lorenzen et al. [12] underscore the aggressive nature of NETs originating from MD, and Modlin et al. [13] suggest that their tendency to metastasize is comparable to, if not surpasses, that of small bowel NETs, in which metastases are present in 64% of patients. Therefore, it is believed that MD NETs should be treated in a manner similar to small bowel NETs rather than appendiceal NETs [11]. According to Nies et al. [14], tumors larger than 5 mm have a marked risk of metastasizing, and by the time symptoms appear, 77% of these tumors have already metastasized. Therefore, aggressive surgical management of tumors larger than 5 mm is recommended by Nies et al. [14]. Sadly, even over a century after the origin of the carcinoid, the size of the primary tumor is most often cited as the critical determinant in the prognosis of its biological behavior [13]. However, metastases can occur with primary tumors that are smaller than 1 cm in diameter; thus, tumor size is an unreliable predictor of metastatic potential [4]. Therefore, the malignant potential of even the smallest lesions should not be overlooked. Evidence of invasive growth and the presence of regional or distant metastases appear to be the best aggregate indicators of prognosis and malignancy [13].

In a study by Soltero and Bill in 1976 [15], the likelihood of MD causing disease over a person's lifetime was estimated to be 4.2%, with the likelihood decreasing with age and the number needed to treat 800. However, the mortality and morbidity rates (7% and 12%, respectively) associated with elective prophylactic removal of normal-appearing MD in adults were discouraging. However, a more recently conducted epidemiological population-based study by Cullen et al. at the Mayo Clinic in Minnesota found that incidental diverticulectomies had a much lower cumulative rate of long-term complications compared to diverticulectomies performed for complications [16]. The incidence of complications requiring surgery was 6.4%, with no age-related trend, and the operative mortality and morbidity rates were lower with incidental diverticulectomy. The mortality rate of these patients was 1.5%, with 7% morbidity; incidental removal had 1% mortality and 2% morbidity. Based on these findings, they concluded that incidental diverticulectomy was appropriate and warranted. Further studies have also shown the safety of diverticulectomy performed incidentally, revealing mortality rates that are near zero or zero [1, 3, 9]. Park et al. analyzed 1,476 patients with MD and found no complications or deaths related to diverticulectomy [17]. Although they did not expressly endorse incidental diverticulectomy, their stance leaned towards a selective approach for resection of incidental MD based on the presence of any of four factors, such as age below 50 years, male sex, diverticulum length exceeding 2 cm, and presence of histologically abnormal tissue, which were statistically significant in predicting complications related to MD.

Additionally, our case adds further evidence to support the claim set forth by Lorenzen et al. that incidental MDs found during surgeries should be removed due to the potential of them containing tumors [12]. In their studies, they also found that despite many of their patients having negative margins of their tumors, their patients still had a high rate of metastasis, with most of their patients showing symptoms of carcinoid tumors or evidence of growth on CT scans. Lorenzen et al. state the importance of palpating the small bowel to look for multicentric tumors (as seen in two of their six patients), as well as perform small bowel resection and lymphadenectomy when the tumor is likely to be larger, and therefore simple diverticulectomy is considered to be inadequate treatment [12]. In Lorenzon et al.'s study [12], only two of the five patients that demonstrated evidence of metastatic disease on CT imaging at the time of diagnosis were the primary tumor identified on imaging. This underscores the significance of considering a tumor originating from the small bowel (or MD) in patients who exhibit liver metastases due to NETs, particularly when a primary tumor cannot be localized using pre-operative imaging. Given that MDs can be challenging to detect on CT scans and many are clinically asymptomatic, we speculate that by leaving incidental MDs unresected when found during surgery, we may be underdiagnosing tumors in these patients, potentially allowing the neoplasm to metastasize and resulting in poorer outcomes.

Thirunavukarasu et al.’s study performed in 2011 endorsed an oncological perspective that appeared to favor resection of incidental MD [3]. The study found that advancing age, particularly above 50 years, increased the risk of developing MDC. Additionally, the incidence of MDC in females has increased significantly over the past few decades. The study suggested that malignancies within MD are often localized, suggesting a high likelihood of curative resection and long-term survival. Therefore, resecting asymptomatic MDs during incidental intraoperative discovery is the most prudent course of action since a preoperative diagnosis of any MD-related pathology is rare. It can be inferred that performing resection when MD is incidentally found during surgery not only eliminates MDC from future diagnoses but also complications of MD, such as persistent fistulas or fibrous band formations. Furthermore, the use of minimally invasive techniques suitable for performing Meckel’s diverticulectomy, such as the recently emerged single incision laparoscopic surgery (SILS), coupled with negligible complication rates in cases of incidental diverticulectomy, supports this approach. Also, robotic-assisted laparoscopy and laparoscopic-assisted resection are other methods found to be particularly useful in complex cases where meticulous dissection is needed, offering less postoperative pain, shorter hospital stays, and quicker recovery compared to other more invasive techniques. Therefore, this implies that the advantages of removing this cancer-prone region surpass the associated risks.

It has been advised that the decision to resect an asymptomatic MD should be individualized for each case. Considerations that would favor resection include younger age at presentation, narrow diverticular neck, history of abdominal adhesions or obstructions, and any palpable or visual abnormality of the MD [2].

To develop evidence-based algorithms for determining when incidental asymptomatic MD should be resected, further research should examine the different criteria associated with neuroendocrine tumors in MDs. Serotonin appears to be the most useful serum biomarker in patients with Meckel's NETs, according to Poncet et al. [10], being elevated in patients tested with Meckel’s NETs. Additionally, Mamikunian et al. suggest that neurokinin A, a biomarker for midgut NETs, is valuable in predicting poor prognosis when the absolute value exceeds 50 pg/ml [18]. This raises the question of whether these biomarkers should be used in the screening and diagnosis of MD and for the prognosis and postoperative monitoring of potential metastasis and adverse outcomes.

## Conclusions

Most NETs in the GI tract are slow-growing and well-differentiated, often leading to delayed diagnosis and advanced disease at the time of diagnosis. Consequently, the best approach for treating incidental MD tumors is through resection due to negligible operative mortality and the high likelihood of curative resection. In our presented patient, removal of the incidentally found asymptomatic MD likely improved the overall medical outcome than if it had not been removed. With the advent of laparoscopic and robotic techniques lowering complication rates, it is recommended to perform prophylactic resection to avoid the risk of developing disease arising from the diverticula.

The decision to perform an incidental MD should be based on the patient's individual risk of developing tumor-related symptoms. It is imperative that clear guidelines be established regarding the management of both symptomatic and incidental asymptomatic carcinoid tumors to prevent metastasis and unfavorable outcomes.
